# Supplementary material for: Perceived Facilitators and Barriers to Nigerian Nurses’ Engagement in Health Promoting Behaviors: A Socio-Ecological Model Approach
Source: Int J Environ Res Public Health. 2020 Feb 18;17(4):1314. doi: 10.3390/ijerph17041314 (PMC7068510; doi:10.3390/ijerph17041314)
Supplement: Supplementary file 1 [file ijerph-17-01314-s001.pdf]

Supplementary file 1: Interview guide for semi-structured interviews on perceived facilitators and barriers to Nigeria nurses engagement in health promoting behaviors (HPBs).

INTRODUCTORY QUESTIONS
*** Think about your daily activities, how would you describe your _______ behaviours?
Probes: *Ask specifically for*

- *Dietary behaviours*
- *Physical activity behaviors*
- *Alcohol consumption behaviors*
- *Smoking behaviours*

Ask about above mentioned behaviors

- *At work*
- *Outside of work*

(If engaging in healthy behaviours) ask the following question for the healthy eating and physical activity.

***Can you explain to me how regularly or frequently you engage in healthy eating/physical activity?

FACILITATORS FOR NURSES ENGAGEMENT IN HPBs

***Think about the healthy choices you mentioned that you make in _____ (named healthy behaviour). Can you tell me about the factors that encourage you to make the healthy choices in ______ behaviours?
Probes:
Ask specifically for facilitators for

- *healthy eating*
- *Physical activity*
- *Non-risky alcohol behaviours*
- *Non-smoking behaviours*

(adapt as applicable to respondent)

***Can you explain how _____ factor (named facilitator) positively affects you?
Probes:
Ask specifically for facilitators for

- *healthy eating*
- *Physical activity*
- *Non-risky alcohol behaviours*
- *Non-smoking behaviours*

(adapt as applicable to respondent)

Ask about facilitators

- *At work*
- *Outside of work.*

*** Tell me of anything you feel would further encourage you (or make it easier for you) to adopt healthy behaviours?
Probes:
Ask specifically for

- *healthy eating*
- *Physical activity*
- *Non-risky alcohol behaviours*
- *Non-smoking behaviours*

(adapt as applicable to respondent)

Consider facilitators:
- *At work*
- *Outside of work.*
-

BARRIERS TO NURSES ENGAGEMENT IN HBPs.

***Think about the health promoting behaviours (Probe: Mention specific HPB as applicable) you previously said you were not practicing (adequately/regularly). Could you describe what you feel prevents you from engaging in ____ (named behaviour(s))?
Probes:
Ask specifically for
- *healthy eating*
- *Physical activity*
- *Non-risky alcohol behaviours*
- *Non-smoking behaviours*

Ask about above mentioned health promoting behaviours
- *At work*
- *Outside of work.*

***How would you explain the challenges you encounter (or mentioned) regarding taking part in _______ (Probe: Mention specific HPB as applicable).
Probe: How does the barrier make it difficult or impossible to make healthy choices sometimes or regularly?

***Can you tell me about any changes you perceive would help to eliminate or reduce the impact of (named barrier)?
Probe*: Repeat question but adapt to elicit information for other barriers mentioned by respondent.*

***Suppose ____ (named barrier) is reduced or removed, do you feel it would improve your engagement in _____ (named HPB)? In what ways would your behaviours in ____ (named HPB) be improved?

*** What sort of programs or interventions do you feel might be helpful for improving your behaviours related to ______ (Probe: Mention specific HPB as applicable)?

CLOSING QUESTIONS
***Would you like to provide any additional information you feel might be relevant that we have not discussed in the course of this interview?
